# Electroacupuncture and Moxibustion Regulate Hippocampus Glia and Mitochondria Activation in DSS-Induced Colitis Mice

**DOI:** 10.1155/2020/2530253

**Published:** 2020-01-23

**Authors:** Ning Zhang, Qun Zhang, Lushuang Xie, Chenyu Li, Zhiqi Zhuang, Sirui Lin, Peiran Lv, Yi Liu, Qiaofeng Wu, Shuguang Yu

**Affiliations:** ^1^Acupuncture and Moxibustion College, Chengdu University of Traditional Chinese Medicine, Chengdu, Sichuan 610075, China; ^2^College of Basic Medicine, Chengdu University of Traditional Chinese Medicine, Chengdu, Sichuan 610075, China; ^3^Institute of Acupuncture and Homeostasis Regulation, Chengdu University of Traditional Chinese Medicine, Chengdu, Sichuan 610075, China; ^4^Department of Rehabilitation, People's Hospital of Pengzhou, Chengdu, Sichuan 610075, China; ^5^Department of Neurology, Dalian Municipal Central Hospital, Affiliated Hospital of Dalian Medical University, Dalian 116033, China

## Abstract

**Objectives:**

To study the influence of electroacupuncture (EA) and moxibustion on the hippocampus astrocyte and microglia activation in the ulcerative colitis model and to evaluate the mitochondria activity.

**Methods:**

2.5% dextran sodium sulfate-induced colitis mice were treated by EA or moxibustion. Intestinal pathological structure was observed by hematoxylin and eosin (H&E) staining; the expression of GFAP or S100b (markers for astrocyte), Iba-1 (a marker for microglia), and Mitofilin (a marker for mitochondria) in hippocampus was detected by immunofluorescence staining or western blot.

**Results:**

The results demonstrated that both EA and moxibustion could improve the morphology of distal colonic mucosal epithelia in DSS-induced colitis mice. Expression of GFAP in the hippocampus was significantly increased after EA or moxibustion treatment. The effects were further supported by WB results. Meanwhile, expression of mitofilin in the hippocampus CA1 and CA3 regions showed the same trend as that of GFAP. Expression of Iba-1 in the hippocampus showed no significant difference after EA or moxibustion treatment, while the state of microglia changed from resting in control mice to activated state in colitis mice.

**Conclusion:**

EA and moxibustion were able to modulate the activation of astrocyte, microglial, and mitochondria in the hippocampus area in the colitis model.

## 1. Introduction

Inflammatory bowel disease (IBD), which is mainly comprised of Crohn's disease and ulcerative colitis, has become a global disease with accelerating incidence in the newly industrialized countries [1]. Despite the incidence looks more stable in the western world, the prevalence of IBD continues to rise [1]. Due to the change of lifestyle, it has an emerging trend in the Eastern Asia countries recently, such as China, Malaysia, particularly a 10-fold increased incidence of IBD over two decades in South Korea [2–4]. The incidence peaks of IBD are in early adulthood, but a large subset of patients that suffer from early childhood through adulthood [5] and the illness permanently affect the quality of life and ability to work. Therefore, it is important to pay more attention to IBD.

Although IBD is a kind of gastrointestinal disease, characterized by chronic intestinal inflammation, dysregulated immune responses to intestinal microbiota, and dysfunction of the epithelial barrier [6], several studies have shown the presence of neuropsychiatric manifestations, such as depression and cognitive dysfunction, particularly during the active stage of the disease [7–10]. In a previous study, we also observed that DSS-induced colitis was accompanied with anxiety behavior [11]. Current theories demonstrated that intestinal inflammation might be a risk factor for the development of ischemic stroke [12], Parkinson's disease (PD) [13], and Alzheimer's disease [14]. It has suggested that gut-derived CD4+ T cells may interact with meningeal macrophages and result in non-gut-derived CD4+ T lymphocyte infiltration into the brain in ischemic stroke [12]. Mild gut inflammation accelerates *α*-synuclein accumulation in PD. Furthermore, most markers of inflammation were elevated in the colon and the brain but not in the blood in DSS-treated PD mice [13]. Similarly, in pathological conditions, such as IBD and PD, the dysregulation of norepinephrine, epinephrine, dopamine, and serotonin neurotransmitters levels can lead to a variety of gastrointestinal symptoms [15]. Also, rats with colitis showed increased hippocampal excitability and cortical inflammation [7, 14, 16]. In acute DSS-induced colitis mice, the expression of IL-6, TNF-*α* elevated, and microglia was activated in the brain tissue. Additionally, the expression of IL-6 and IL-1*β*, cyclin-dependent kinase inhibitor p21Cip1 (p21), cyclo-oxygenase 2 (COX-2), and glial fibrillary acidic protein (GFAP) was increased in hippocampus. Briefly, peripheral inflammation is likely to explain at least some of the neurobehavioral symptoms associated with chronic inflammatory diseases. However, works with regard to the links between IBD and hippocampus glial cell changes are largely unknown.

Hippocampus is highly susceptible to damage of hypoxia, ischemia, or encephalitis. Raghavendra et al. found that CFA-induced peripheral inflammation leads to robust glial activation at the lumbar spinal cord, brainstem, and forebrain. Significantly elevated microglial activated markers (Mac-1, TLR4, and CD14) were expressed during acute, subacute, and chronic phases. And upregulated astroglia markers (GFAP and S100B) were expressed during subacute and chronic phases of the inflammation [17]. Astrocytes and microglia are glial cells sensitive to changes in the microenvironment, especially inflammation. It was found that profound synaptic changes resulted from a mirror microglia-mediated inflammatory response in hippocampus and in the colonic inflammation model [9]. In the TNBS-treated gut inflammation, rats exhibited a marked, reversible inflammatory response within the hippocampus, characterized by microglial activation and increases in TNF-*α* levels [18]. Additionally, mitochondria are the powerhouse of the cell due to the central role in metabolism and energy production. Activation of mitochondria demonstrated more energy support for astrocytes and neurons. Moreover, mitochondria have been implicated in inflammation, cell death, and senescence [19]. Therefore, it could be hypothesized that peripheral inflammation has a crucial influence on the activation of central glial cells and mitochondria under neuroinflammation.

Clinical and experimental studies have shown beneficial role of acupuncture and moxibustion in reducing IBD disease activity and inflammation [20–22]. Our previous studies also found that both EA and moxibustion have therapeutic effect on colitis rats [4, 11, 23]. Herein, we sought to observe whether EA and moxibustion could be able to regulate the astrocyte and microglial and mitochondria activation of hippocampal area in the colitis model, providing new clues for studying the curative effect of EA and moxibustion on the basis of gut-brain axis.

## 2. Materials and Methods

### 2.1. Animals

**Figure 1:**
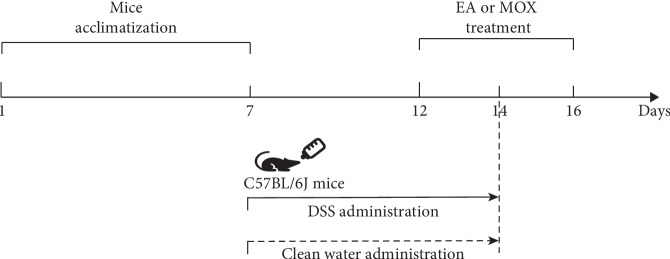
Schematic diagram for the experimental procedure. Horizontal black arrow line represents the whole experimental procedure and the key time points. The solid arrow represents the oral administration of 2.5% DSS in drinking water to induce colitis until the 14^th^ day, and the dotted arrow represents drinking clean water as a control group. The EA or MOX treatment procedure applied to mice was started from the 5^th^ day of DSS water administration. DSS, dextran sodium sulfate; EA, electroacupuncture; MOX, moxibustion.

A total of 24 male C57BL/6J mice (6–8 weeks) were used. Mice were housed under constant room temperature (20 ± 2°C) and humidity (50–60%) with a 12 : 12 h light/dark cycle, and they were free to access water and food. After one week's adaptation, the mice were randomly divided into 4 groups: the control group, the colitis group, the EA group, and the moxibustion group. The colitis model was induced by drinking dextran sodium sulfate (DSS, 43 kDa, MP Biomedicals) water as previously described [24, 25]. The survival rate and the evaluation standard of colitis model were the same as we reported before [26]. At the 5^th^ day of DSS administration, the successful colitis-induced mice were checked and confirmed by fecal occult blood and the pathological morphology of the gut. The administration of DSS water to mice would last for 7 consecutive days totally. Meanwhile, mice in the control group received normal drinking water (Figure 1).

The experimental animals were purchased from Sichuan Dashuo Experimental Animal Co. Ltd. (license number: SCXK (chuan) 2015-030). Animal care, experimental procedures, and operations carried out in the present study were guided by the Animal Care and Use Committee of Chengdu University of Traditional Chinese Medicine. The ethics number for this experiment is (2014-07).

### 2.2. Electroacupuncture and Moxibustion Treatments

The EA and moxibustion applied to mice were started at the 5^th^ day of DSS water administration when the mortality is the lowest proved by the previous study [26]. The acupoints of Guanyuan (CV4) and Zusanli (ST36) were used, which were effective for treating UC according to the clinical practice and our previous studies [27, 28]. In the EA and moxibustion groups, acupoints CV4 were administered every day, while bilateral ST36 was alternately used. The locations of these acupoints were determined according to Government Channel and Points Standard GB12346-90 of China and “the Veterinary Acupuncture of China.” EA was performed with a 0.25 mm needle with a length of 13 mm being introduced to a depth of 3 mm. The acupuncture procedure was manipulated with a connected Hans-200. Acupoint nerve stimulator to provide a sparse dense wave at a frequency of 2/15 Hz for 15 min each day and the period of care lasted for 5 days. The moxa sticks (0.5 cm diameter; 15 cm length) were hung 1.5 cm above CV4 and ST36. The moxa stick (diameter × length: 0.4 × 10 cm, Nanyang Hanyi Moxibustion Technology Development Co. Ltd., China) was burned to carry out moxibustion over ST36 and CV4 for 15 min per day and lasts for 5 consecutive days. For moxibustion and EA treatment, the mice were fastened on the special fixer (self-designed) on a supine position. Mice in the control and colitis groups were restricted for 15 min on the same fixer without any treatments.

### 2.3. Sample Collection

Mice were firstly anesthetized by 1% pentobarbital sodium (3 mL/kg) and then killed by cervical dislocation. Secondly, the hippocampus and colon tissue for western blot was removed quickly on ice and then stored at −80°C. Thirdly, the colon tissue for hematoxylin-eosin or immunofluorescence was removed, cut open, cleaned with precooling normal saline, and immersed and fixed in 4% paraformaldehyde solution.

### 2.4. Histological Observation

The intestinal tissues stored at 4°C were taken and dehydrated in an alcohol series. The paraffin-embedded colon was cut into sections (thick 3–5 *μ*m), sections were deparaffinized by xylene, and xylene was removed by alcohol. Then tissue sections were stained with H&E and the morphology of distal colonic mucosa was observed under the electron microscopy (Mike Audi BA200Digital).

### 2.5. Immunofluorescence Staining

To evaluate the activation of astrocyte and microglia, the expression of GFAP and Iba-1 was detected in the hippocampal CA1, CA3, and DG regions by immunofluorescence. To evaluate the activity of mitochondria, the expression of mitofilin was also tested. Briefly, brain tissues were fixed in 4.0% buffered paraformaldehyde, embedded in paraffin, and sectioned into 20 *μ*m thick slices. Sectioned samples were deparaffinized in xylene, rehydrated in a series of graded alcohol, and subjected to antigen retrieval. Endogenous peroxidase was quenched with 3.0% hydrogen peroxide in methanol for 30 min. Sections were further blocked with 3.0% bovine serum albumin (BSA) in PBS, exposed to 0.5% Triton X-100 for 1 h for reducing nonspecific antibody binding and incubated with mouse GFAP (Abcam. Inc., USA), rabbit mitofilin (Abcam. Inc., USA), and goat Iba-1 (Bioss, China) antibody (1 : 200) at 4°C overnights. The sections were washed with PBS three times and incubated with secondary antibody (Alexa Fluor 488, cy3, Bioss, China) (used at 1 : 200 dilution) for 120 min at 37°C. Five random fields (200x) were counted in each sectioned sample by a researcher blinded to the treatment. The relative % area positive expression was quantified by the Image-Pro Plus 6.0 (Media Cybernetics, USA).

### 2.6. Western Blot

Western blot analysis was performed as described in [4]. Protein extracts were prepared from the mice hippocampus using a lysis buffer supplemented with ethylenediaminetetraacetic acid- (EDTA-) free complete protease inhibitors. Proteins were extracted and subjected to sodium dodecyl sulfate polyacrylamide gel electrophoresis (SDS-PAGE) on 10% gel. The bands were transferred to a polyvinylidene difluoride membrane. After blocking with 5% nonfat dry milk in Tris-buffered saline supplemented with 0.1% Tween 20 for 4 h at room temperature, the membranes were incubated overnight at 4°C with the following antibodies: S100b (Bioss, China) primary antibodies (used at 1 : 800 dilution). The membranes were then incubated with a secondary antibody at 37°C for 1 h. Normalization was performed by blotting the same membranes with anti-*β*-actin antibody (Abcam. Inc. USA). All western blot data were analyzed by Image-Pro Plus 6.0 software.

### 2.7. Statistical Analysis

All data were presented as the mean ± standard error of mean (mean ± SEM) and evaluated by one-way ANOVA performed using GraphPad Prism 7 (GraphPad Prism Software Inc., San Diego, USA). The level of significance was set at *P* < 0.05.

## 3. Results

### 3.1. EA and Moxibustion Alleviate DSS-Induced Colitis in Mice

**Figure 2:**
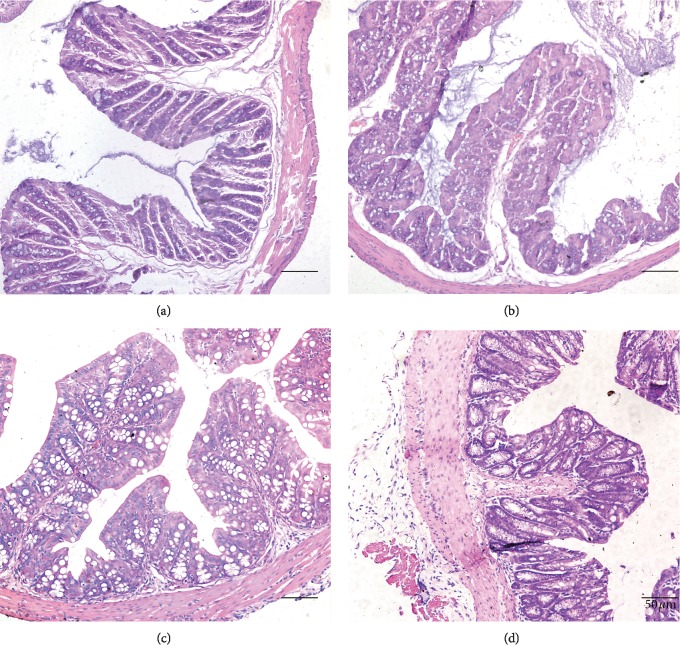
Morphological changes in different groups. Leukocyte infiltration into the mucosa and damage to the colon wall were evident in the DSS-induced colitis group (H&E, 200x). (a) Con, control group; (b) DSS, DSS-induced colitis group; (c) DSS + MOX, moxibustion treatment group; (d) DSS + EA, electroacupuncture treatment group.

Results of histological analysis showed that the colons from the DSS-treated mice expressed obviously damaged mucosa and loss of gland and inflammatory cell infiltration. After EA and moxibustion treatments, the histopathological conditions of mice in the treatment group have been improved, as evidenced by neatly arranged epithelial mucosa and glands as well as the disappearance of most of the infiltrating inflammatory cells (Figure 2).

### 3.2. EA and Moxibustion Promote Astrocyte Activation

**Figure 3:**
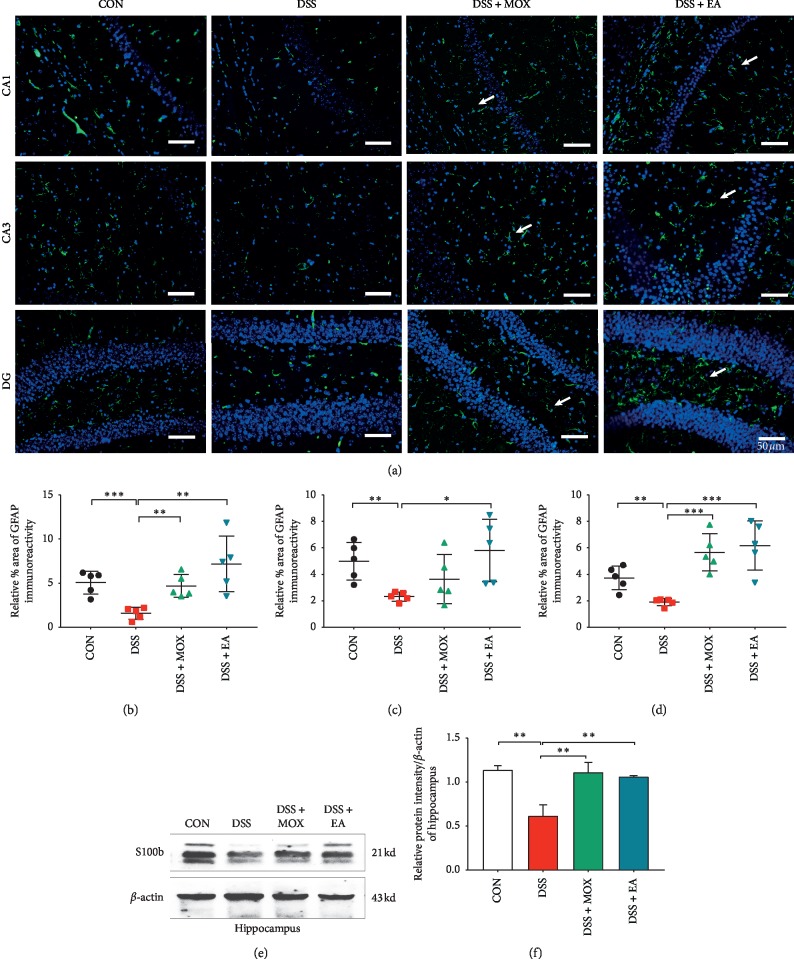
EA and moxibustion promote astrocyte activation. Labeling (a) and analysis of astrocytes in the hippocampus CA1 (b), CA3 (c), and DG (d) regions of UC mice. Green, GFAP (a marker for astroglia); blue, DAPI nuclear staining (*n* = 5/group). White arrow highlights the activated astrocyte. Scale bar, 50 *μ*m. (e, f) Western blot (e) and analysis (f) showing the downregulation of S100b (a marker for astroglia) expression in the hippocampus in the DSS-induced colitis group and upregulation after EA and moxibustion treatment (*n* = 3/group). GFAP, glial fibrillary acidic protein.

The GFAP and S100b positive cells were marked and are shown in Figure 3; compared with control mice, the expression of GFAP was significantly decreased after DSS-induced colitis. The results showed that the % area of GFAP positive expression of hippocampus CA1 (Dss vs Con, 1.58 ± 0.31 vs 5.10 ± 0.59, *P* < 0.001), CA3 (2.34 ± 0.15 vs 5.04 ± 0.64, *P* < 0.01), and DG (1.91 ± 0.12 vs 3.76 ± 0.40, *P* < 0.01) (Figure 3) regions in the model group had statistical significance. However, the % area of GFAP positive expression markedly increased after EA (Dss + EA vs Dss, CA1: 7.25 ± 1.43 vs 1.58 ± 0.31, *P* < 0.01; CA3: 5.86 ± 1.07 vs 2.34 ± 0.15, *P* < 0.05; DG: 6.25 ± 0.83 vs 1.91 ± 0.12, *P* < 0.001) and moxibustion (Dss + Mox vs Dss, CA1: 4.73 ± 0.59 vs 1.58 ± 0.31, *P* < 0.01; DG (5.72 ± 0.63 vs 1.91 ± 0.12, *P* < 0.01) treatment, except for the expression of GFAP in hippocampus CA3 area after moxibustion treatment (3.67 ± 0.84 vs 2.34 ± 0.15, *P* > 0.05). Moreover, there was no obvious difference between EA and moxibustion groups (*P* > 0.05) (Figure 3). Western blot results showed that the expression of S100b in the hippocampus was also upregulated after EA (Dss + EA vs Dss, 1.07 ± 0.005 vs 0.62 ± 0.07, *P* < 0.01) and moxibustion (Dss + MOX vs Dss, 1.12 ± 0.07 vs 0.62 ± 0.07, *P* < 0.01) treatment.

### 3.3. EA and Moxibustion Modulate Microglia Activation

**Figure 4:**
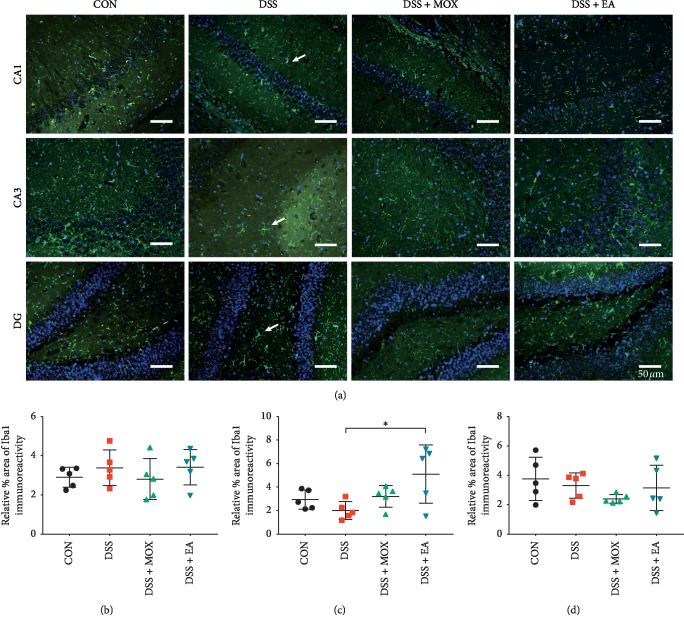
EA and moxibustion modulate microglia activation. Labeling (a) and analysis (b–d) of microglia in the hippocampus CA1 (b), CA3 (c), and DG (d) regions of UC mice. Green, Iba-1 (a marker of activated microglia); blue, DAPI nuclear staining (*n* = 5/group). Scale bar, 50 *μ*m. White arrow highlights the state change of microglia, from resting in control mice (faint Iba-1 staining) to activation in UC mice (DSS-induced; strong Iba-1 staining) and also improvement after EA and moxibustion treatment. Iba1, ionized calcium binding adapter molecule 1.

To evaluate brain inflammation in this model, we performed immunofluorescence staining for Iba-1 and analyzed the microglial population in the hippocampus CA1, CA3, and DG regions. Compared with control group, the % area of Iba-1 positive expression of hippocampus CA1 (Dss vs Con, 3.42 ± 0.41 vs 2.92 ± 0.23, *P* > 0.05), CA3 (2.02 ± 0.35 vs 2.97 ± 0.37, *P* > 0.05) and DG (3.33 ± 0.39 vs 3.79 ± 0.67, *P* > 0.05) showed no significant difference after DSS-induced colitis (Figure 4). But, the state of microglia changed from resting in control mice to activation in UC mice (Figure 4(a)).

Additionally, there was no obvious difference after EA (Dss + EA vs Dss, CA1: 3.45 ± 0.41 vs 3.42 ± 0.41, *P* > 0.05; DG: 3.18 ± 0.69 vs 3.33 ± 0.39, *P* > 0.05) and moxibustion (Dss + Mox vs Dss, CA1: 2.83 ± 0.47 vs 3.42 ± 0.41, *P* > 0.05; CA3: 3.23 ± 0.41 vs 2.02 ± 0.35, *P* > 0.05; DG (2.02 ± 0.29 vs 3.33 ± 0.39, *P* > 0.05) treatment. However, after EA treatment, compared with the model group, the % area of Iba-1 positive expression of hippocampus CA3 had a significant elevation (Dss + EA vs Dss, 5.15 ± 1.12 vs 2.02 ± 0.35, *P* > 0.05).

### 3.4. EA and Moxibustion Promote Mitochondria Activation

**Figure 5:**
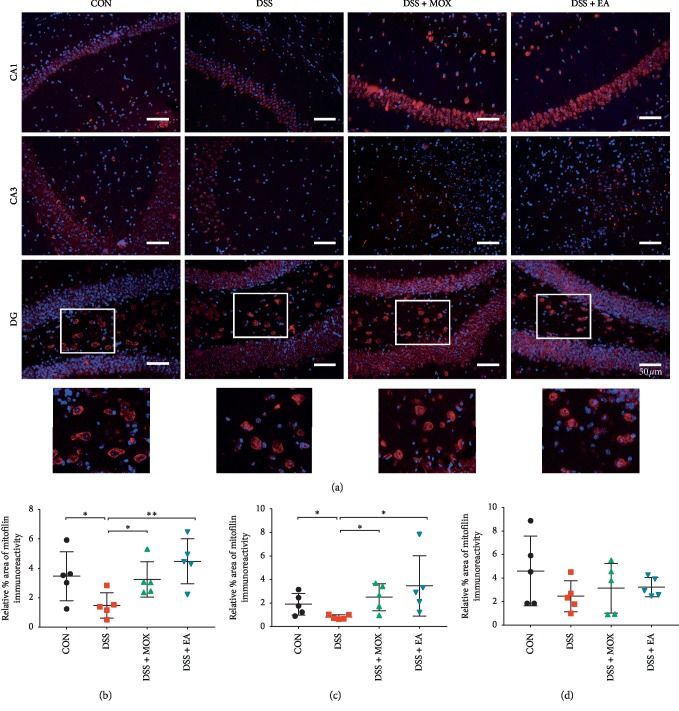
EA and moxibustion promote mitochondria activation. Representative labeling (a) and analysis (b–d) of mitochondria in the hippocampus CA1 (b), CA3 (c), and DG (d) regions of UC mice. Mitofilin (a marker of activated mitochondria); blue, DAPI nuclear staining (*n* = 5/group). Scale bar, 50 *μ*m. Below: enlarged views of the white frames in the above panels highlight the cytoplasmic positive expression of mitochondria in the DG region of hippocampus.

Results of activated mitochondria in the hippocampal CA1 and CA3 regions showed the same trend as that of GFAP and that the % area of mitofilin positive expression from DSS-induced colitis mice decreased significantly compared with control mice CA1 (Dss vs Con, 1.50 ± 0.39 vs 3.50 ± 0.76, *P* < 0.05) and CA3 (0.82 ± 0.08 vs 1.92 ± 0.41, *P* < 0.05), as shown in Figure 5. Furthermore, the % area of mitofilin positive expression in both colitis + moxibustion (Dss + Mox vs Dss, CA1: 3.28 ± 0.54 vs 1.50 ± 0.39, *P* < 0.05; CA3: 2.52 ± 0.52 vs 0.82 ± 0.08, *P* < 0.05) and colitis + electroacupuncture (Dss + EA vs Dss, CA1: 4.52 ± 0.61 vs 1.50 ± 0.39, *P* < 0.01; CA3: 3.51 ± 1.16 vs 0.82 ± 0.08, *P* < 0.05) mice increased markedly compared with colitis mice.

However, the % area of mitofilin positive expression in the hippocampal DG region showed no difference among control, DSS-induced colitis, colitis + moxibustion, and colitis + electroacupuncture mice (*P* > 0.05). Surprisingly, the positive expression of mitochondria in DG region of hippocampus showed cytoplasmic positive.

## 4. Discussion

In this study, we found that the therapy of EA and moxibustion on colitis mice can affect the hippocampus inflammation by activation of astrocyte and microglial. We also found that mitochondria, the center of energy metabolism, were enhanced by the above treatments.

The brain-gut axis has become an important theoretical basis for treating both brain and gastrointestinal diseases. Peripheral inflammation could affect the central nervous system (CNS) through inflammation cytokines and vagus nerve. Accordingly, brain diseases can also cause gastrointestinal symptoms [9]. DSS-induced colitis is a typical peripheral inflammation. It is reported that colitis significantly exacerbated microstroke in mice, and vagus nerve stimulation alleviated colitis-induced cerebral cortical microinfarct aggravation via decreased blood-brain barrier (BBB) permeation, resulting in microglia and astrocytes activation, oxidative stress, and proinflammatory cytokine expression [29]. Fan et al. found that the limbic lobe might be the core of the brain-gut axis and play an important role in cognitive impairment in patients with colitis during the active stage [8]. Nevertheless, the study has shown that the cytokines generated during peripheral inflammation activate a secondary, mirror inflammatory response in the brain that was characterized by activation of microglia and production of proinflammatory cytokines [9]. In this study, we found that Iba-1-positive cells activated in the hippocampus of UC mice indicating the activation of microglia. The potential reasons would be that the inflammatory reaction in the brain is directly caused by peripheral inflammation or secondary inflammation occurrences when signals from the gut arrive in CNS. Astrocytes, another important glia, were also been found involving in this processing. Astrocytes play a significant role in maintaining neuronal and vascular networks in CNS. It takes part in many kinds of activity in the brain and spinal cord. Reactive proliferation of astrocytes would occur during CNS injury. It provides some protection by secreting anti-inflammatory factors, migrating to the injury area and formulating astrocytic scar [9]. Various studies have shown that mitochondria are also closely related to neuroinflammation [30, 31]. In DSS-induced colitis mice, mitochondria-targeted antioxidant could inhibit the barrier dysfunction and suppressed colitis in the DSS-induced model [30]. In the senescence animal model, impairment in learning and memory skills was partly due to brain mitochondrial DNA damage and repair capacity defection [32]. Neuroinflammation can decrease *α*7 nicotinic acetylcholine receptor expression in both mitochondria and cell plasma membrane in the brain of mice and makes mitochondria more susceptible to apoptosis induction [33]. Further enhancing neurogenesis and inhibiting inflammation and oxidative stress through normal mitochondrial respiration may be the basis for better memory and emotional function [34]. Therefore, we assume that peripheral inflammation may also have some influence on the activation of mitochondrial under neuroinflammation.

In this study, the decrease of GFAP expression in the model group was initially unexpected. However, it has been established that astrocytes are activated during inflammation [35]. Nevertheless, evidence of reduced GFAP immunoreactivity following traumatic brain injury (TBI) has been reported previously and is attributed to the breakdown of intermediate filaments and a concomitant alteration in overall protein function [36]. In light of TBI inducing glial activation and neuroinflammation in the hippocampus, it is possible that similar mechanism could underlie the reduced GFAP observed in the hippocampus in DSS-induced colitis model mice [37]. Furthermore, the decrease of astrocyte in the model group may be related to the suppression of anti-inflammatory effect of hippocampal astrocyte; on the contrary, both EA and moxibustion could activate the GFAP expression in the hippocampus and contribute to the therapeutic effect on hippocampus inflammation. Taking the results of other people's research into account, it might be due to the different stages of the disease. For instance, Svetlana et al. demonstrated that, during the chronic phase of IBD, GFAP was induced [14]. The study has shown that EA could activate the lactate metabolism in the resident astrocytes around the ischemic area, which facilitated the transfer of intracellular lactate to extracellular domain to be utilized by injured neurons to improve the neurological deficit [38]. Astrocytes can provide many important functions to support neurons, including exchange of metabolic and nutritional material, maintenance of ion concentrations, and clearance neurotransmitters in the vicinity of neuron. Damage to or loss of astrocytes has important implications for neuronal survival and brain function [39]. Thus, it should be one of the mechanisms that EA and moxibustion cure colitis by increasing the activation of astrocytes. On the one hand, the activation of astrocyte would provide some protection to the brain which could decrease the disturbance of CNS. For example, it would prevent the occurrence of psychiatric symptoms. On the other hand, astrocyte could keep the balance of environment of CNS, which helps the brain affect the situation of the gut and intestinal microorganisms.

As presented before, no significant change was found in microglia expression between the model group and treatment groups. However, in the colitis group, the state of microglia turned into activated microglia. Microglial cells are resident macrophages in the CNS, which can monitor the surrounding environment and play important roles in CNS development, maintenance, and disease [40]. Microglia is activated by inflammation, and it also has an inhibitory effect on inflammation. Experimental models of IBD have shown a clear connection between intestinal inflammation and changes in brain function. For instance, electrophysiological recordings of hippocampal slices from animals with chronic intestinal inflammation show enhanced excitability, which may be due to increased TNF-*α* signaling and microglial activation within the brain [18]. During the acute phase of colitis, plasma levels of IL-6 are increased, accompanied by activation of microglia, as well as increased levels of IL-1*β*, TNF-*α*, and p21 in the hippocampus. As p21 is expressed in NPC and in early neuroblasts, it is plausible to consider that cytokine-induced p21 expression might be responsible for the decreased neurogenesis, given that nestin and brain lipid binding protein, both markers of NPC, as well as DCX, an early neuronal marker, are all decreased [14, 41]. Additionally, Han et al. found that DSS-induced colitis could increase the cortical inflammation and trigger the activation of microglia as well as reduction of occludin and claudin-5 expression. That is to say, it could generate a mild brain injury by reducing the tight junction-related protein expression but not by severely increasing BBB permeability [16]. Compelling evidence shows that microglia serves as endogenous regulators of inflammation in the CNS and maintains communication with the peripheral immune system to enable recruitment of peripheral immune cells in case of injury or infection [42]. With the promotion of microglia activation after EA or moxibustion treatment, it may implicate that both EA and moxibustion at the CV 4 and ST 36 acupoints could effectively accelerate the alleviation of CNS inflammation. This could bring some benefits to CNS. Firstly, it decreases the inflammation in CNS, so that it could avoid brain from damage. Secondly, it could restore the normal function of microglia to reduce the inflammation. Studies have reported that acupuncture could alleviate spinal microglial activation to achieve the aim of reducing scratching behavior [43]. Furthermore, our previous study found that EA has such therapeutic effects on Alzheimer's disease mice, by promoting the polarization of microglia cells to neuroprotective M2 phenotype and inhibiting the information of cytotoxic M1 phenotype microglia cells [44].

Interestingly, in the present study, our results showed that activated mitochondria after EA and moxibustion in the hippocampal CA1 and CA3 regions showed same trend as that of GFAP. Mitochondria have traditionally been viewed as the powerhouse of the cell due to their central role in metabolism and energy production. More recently, mitochondria have been implicated in other cellular roles including calcium storage, migration, cell death, senescence, and inflammation [19]. Sustaining mitochondria is one of the important functions of astrocyte in CNS. In addition, neurons are exquisitely dependent upon mitochondrial respiration to support energy-demanding functions. Thus, activation of mitochondria may demonstrate more energy support for astrocytes and neurons. Sun et al. found that acupuncture and moxibustion could recover mitochondria injury and protect mitochondria function via improving the ultrastructure of mitochondria and increasing the levels of SIR1 in AD mice [45]. Furthermore, studies have demonstrated that induction of autophagy in cells can inhibit LPS-induced microglial inflammation [46]. Functional mitophagy may act as a scavenger of mitochondrial reactive oxygen species through the removal of damaged mitochondria and thereby suppresses inflammatory responses of microglial cells [40]. It is possible that a similar mechanism might underlie, however, whether the local mitochondrial expression changed by EA and moxibustion could induce autophagy in cells, and finally suppresses the inflammatory responses of microglial cells and need further investigation.

In traditional Chinese medicine, CV4 and ST36 acupoints are often used to treat gastrointestinal diseases. According to the clinical practice and our previous studies, CV4 and ST36 were effective for treating UC [27, 28]. Several studies have shown that EA and moxibustion can regulate inflammatory mediators and colonic mucosal barrier in UC model rats. For instance, Ma et al. found that moxibustion at CV4 and ST36 acupoints could improve intestinal mucosal tissue repair by reducing TNF-*α* and p38MAPK and increasing the expression of occludin and ZO-1 in colon tissues [47]. Also, EA at ST36 point could regulate the intestinal immune function by modulating IL-1*β* and nAchR*α*7 mRNA and reduce mucosal lesions to achieve the purpose of treating UC [48]. Furthermore, electrical stimulation at ST36 improves the colonic inflammation in TNBS-treated rats by inhibiting proinflammatory cytokines via the autonomic mechanism [49]. Therefore, it is necessary to choose these two points for UC.

Hence, based on the evidence provided in current and previous studies, we found that EA and moxibustion could regulate the local hippocampal astrocyte and microglial and mitochondria activation, which may further produce curative effects such as secreting brain-gut peptides and then alleviate the neurobehavioral symptoms associated with chronic inflammatory diseases. However, our study has some limitations. Firstly, we only used the acute phase of the colitis model, and future studies can take the chronic phase model into account in order to observe different changes. Additionally, given the purported role of glia cells and mitochondrial in CNS, it is necessary to conduct further research studies on the potential mechanisms of EA or moxibustion to alleviate the neurobehavioral symptoms of chronic inflammatory diseases.

## 5. Conclusion

In the present study, EA and moxibustion treatments promote the activity of astrocyte, microglia, and the level of mitochondria in hippocampus in the DSS-induced colitis mice model, which providing a direct evidence that EA and moxibustion can influence the cross-talk between brain-gut axis.

## Data Availability

The data used to support the findings of this study are available from the corresponding author upon request.

## Supplementary Materials

Supplementary Materials Electroacupuncture and moxibustion regulate hippocampus glial cells and mitochondria activation in ulcerative colitis mice. Peripheral inflammation could affect the central nervous system (brain-gut axis). Astrocytes, microglia, and mitochondria play significant roles in the central nervous system homeostasis and disease (blue words). Here, we observed that both electroacupuncture and moxibustion can induce activation of hippocampal astrocytes, microglia, and mitochondria in a colitis model. It may provide new clues for studying the curative effect of electroacupuncture and moxibustion on the basis of gut-brain axis.
