# Supplementary material for: Electroacupuncture and Moxibustion Regulate Hippocampus Glia and Mitochondria Activation in DSS-Induced Colitis Mice
Source: Evid Based Complement Alternat Med. 2020 Jan 23;2020:2530253. doi: 10.1155/2020/2530253 (PMC7003272; doi:10.1155/2020/2530253)
Supplement: Supplementary Materials — Electroacupuncture and moxibustion regulate hippocampus glial cells and mitochondria activation in ulcerative colitis mice. Peripheral inflammation could affect the central nervous system (brain-gut axis). Astrocytes, microglia, and mitochondria play significant roles in the central nervous system homeostasis and disease (blue words). Here, we observed that both electroacupuncture and moxibustion can induce activation of hippocampal astrocytes, microglia, and mitochondria in a colitis model. It may provide new clues for studying the curative effect of electroacupuncture and moxibustion on the basis of gut-brain axis. [file 2530253.f1.pdf]

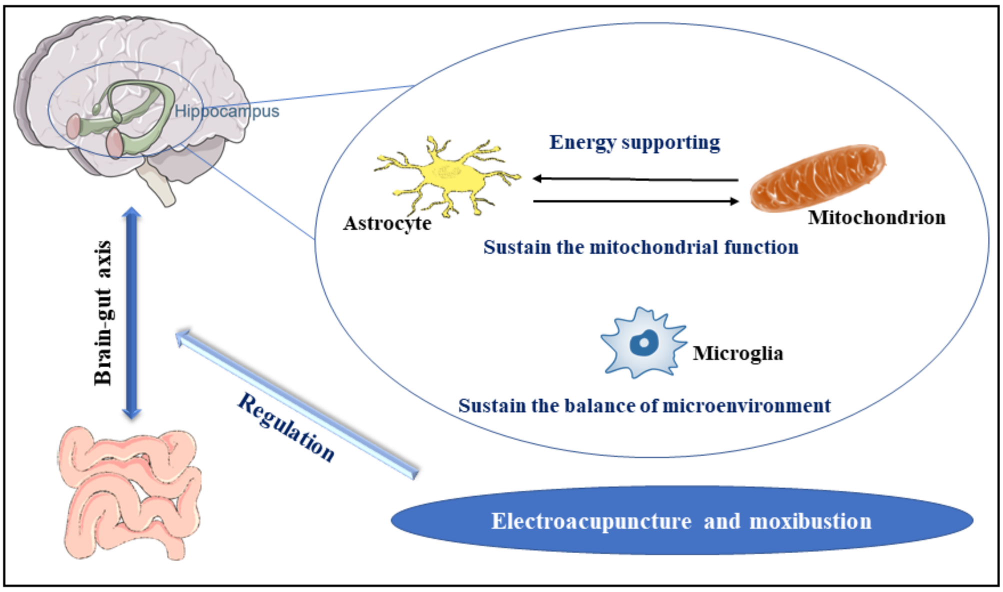

Description of supplementary materials

Electroacupuncture and moxibustion regulate hippocampus glial cells and mitochondria activation in ulcerative colitis mice. Peripheral inflammation could affect central nervous system (brain-gut axis). Astrocytes, microglia and mitochondria play significant roles in central nervous system homeostasis and disease (blue words). Here we observed that both electroacupuncture and moxibustion can induce activation of hippocampal astrocytes, microglia and mitochondria in a colitis model. It may provide new clues for studying the curative effect of electroacupuncture and moxibustion on the basis of gut-brain axis.
